# Ileal Neuroendocrin Tumor Metastasis to Breast Diagnosed with Ga-68 DOTATATE PET/CT

**DOI:** 10.4274/mirt.galenos.2019.28199

**Published:** 2019-09-06

**Authors:** Sevda Sağlampınar Karyağar, Osman Güven, Savaş Karyağar

**Affiliations:** 1 University of Health Sciences, Okmeydanı Training and Research Hospital, Clinic of Nuclear Medicine, İstanbul, Turkey

**Keywords:** Ga-68 DOTATATE PET/CT, well differentiated neuroendocrin tumor, breast metastasis

## Abstract

Breast metastasis of the well differentiated neuroendocrin tumor (WDNET) of the ileum is very rare. A case of a 62-year-old woman with ileal WDNET, who underwent restaging with Ga-68 DOTATATE PET/CT due to progression of metastatic lesions under the treatment with somatostatin analog and mammalian target of rapamycin inhibitors. Ga-68 DOTATATE PET/CT demonstrated intense increased uptake in the subsantimetric nodular lesion in the upper outer quadrant of the left breast. The histopathologic findings obtained by tru-cut biopsy revealed WDNET metastasis (Ki-67 proliferation index 1%).

## Abstract (tr)

İyi diferansiye ileal nöroendokrin tümör (NET) meme metastazı oldukça nadirdir. Altmış iki yaşında iyi diferansiye NET tümör tanılı kadın hastaya, metastatik lezyonlarının somatostatin analoğu ve rapamisinin memeli hedefi inhibitörleri ile tedavi görmekte iken progrese olması nedeniyle yeniden evreleme amacıyla Ga-68 DOTATATE PET/BT görüntüleme yapıldı. Ga-68 DOTATATE PET/BT görüntülemede, sol meme üst dış kadrandaki subsantimetrik nodüler lezyonda yoğun artmış aktivite tutulumu izlendi. Lezyondan yapılan tru-cut biyopsinin histopatolojik incelemesinde iyi diferansiye NET metastazı olduğu tespit edildi (Ki-67 proliferasyon indeksi %1).

## Figures and Tables

**Figure 1:**
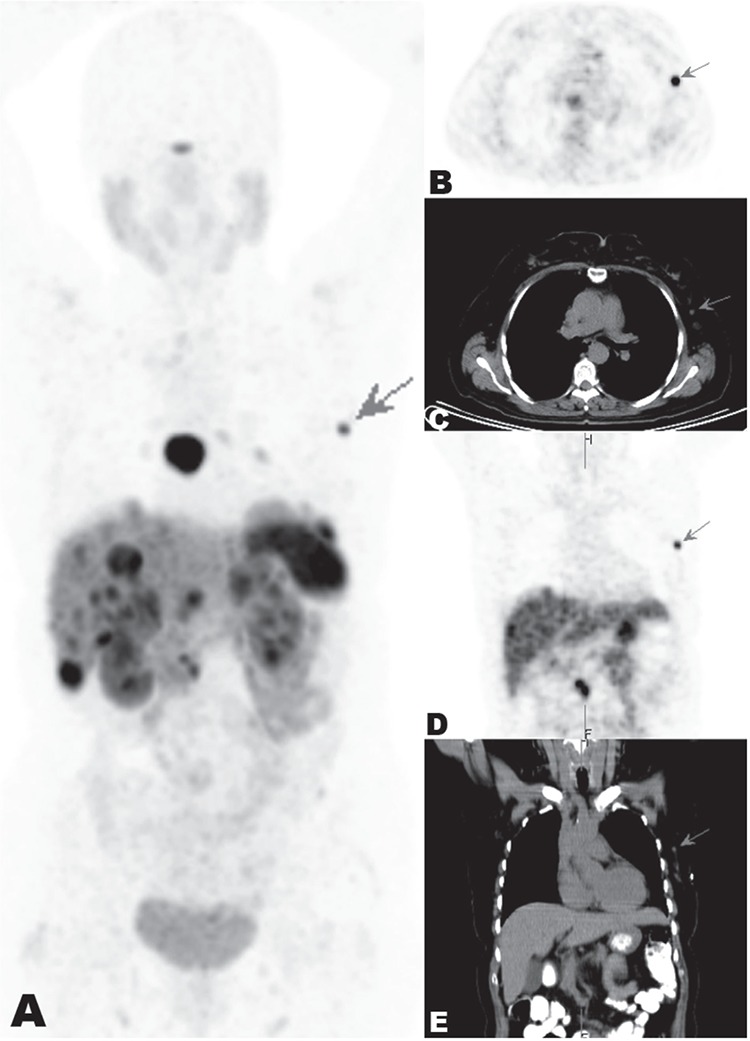
Ga-68 DOTATATE PET/CT images: The whole body coronal image on PET (A), axial images on PET (B) and CT (C) and coronal images on PET (D) and CT (E) at the thoracic level. A 62-year-old woman followed up with well differentiated neuroendocrin tumor (WDNET) of ileum for 6 years was referred for restaging with PET/CT imaging due to progression of metastatic lesions under the treatment with somatostatin analog and mTOR inhibitors. The patient was injected with 5 mCi of Ga-68 DOTATATE intravenously. After 60 minutes of waiting, the patient was imaged from basis of skull to middle of the thigh using an integrated PET/CT scanner which was consisted of a full-ring high resolution PET with lutetium oxy-orthosilicate crystal and a 6-slice CT (Siemens Biograph 6, Chicago, USA). There were multiple hepatic nodular lesions, multiple intense mediastinal (biggest in the subcarinal station) and intraabdominal lymph nodes showing intense somatostatin receptor activity compatible with metastasis. Ga-68 DOTATATE PET/CT images also showed intense increased uptake in the subsantimetric nodular lesion in the upper outer quadrant of the left breast, suggesting metastasis (A, B, C, D and E, arrow). The histopathologic findings obtained by trucut biopsy revealed WDNET metastasis (Ki-67 proliferation index 1%). Ga-68 DOTATATE PET/CT is widely used for initial staging and restaging of WDNET (1). Ileal WDNET usually metastasizes to liver, mesenteric lymph nodes, lung, peritoneum and pancreas (2). Breast metastasis of ileal WDNET is a very rare entity (3,4,5,6). Ga-68 DOTATATE PET/CT can detect metastatic lesions of WDNET in uncommon regions such as breast. Also, diagnosis of breast lesion with Ga-68 DOTATATE uptake on PET/CT lets us distinguish WDNET metastasis from primary breast malignancy without neuroendocrin differentiation. In this case, we report a patient with breast metastasis of ileal WDNET detected with Ga-68 DOTATATE PET/CT.
